# Digital Transformation and Green Innovation of Chinese Firms: The Moderating Role of Regulatory Pressure and International Opportunities

**DOI:** 10.3390/ijerph192013321

**Published:** 2022-10-15

**Authors:** Jinqiu He, Huiwen Su

**Affiliations:** 1 School of Economics, Central University of Finance and Economics, Beijing 100081, China; 2 Renmin Business School, Renmin University of China, Beijing 100872, China

**Keywords:** digital transformation, green innovation, structural contingency theory, regulatory pressure, internationalization

## Abstract

The digitalization of business processes has increasingly challenged conventional wisdom in corporate green innovation. This empirical paper studies the timely but theoretically underexplored relationship between digital transformation and green innovation in a developing country context. Given that firms’ digital transformation shifts organizational structures toward decentralization, we employ a digital perspective to analyze organizational coordination, control, and learning mechanisms and propose that digital transformation positively affects corporate green innovation. Moreover, drawing on structural contingency theory, we demonstrate that such effects can be strengthened by external contingencies, specifically regulatory pressure and international opportunities. Using a dataset of Chinese listed firms, we find empirical support for our hypotheses. Our study is one of the first to examine how firms can leverage organizational digital transformation to enhance their green innovation performance and thus provides new insights into the drivers of sustainable practices for firms in developing countries.

## 1. Introduction

Green innovation has been proven to be a crucial factor in achieving economic profitability and environmental sustainability [1,2,3]. Given the fragile institutional environment and heterogeneous firm capabilities, environmental scholars call for a uniform paradigm that addresses both firms’ structural change and green innovation [4,5]. Related literature has long been dominated by the resource-based view and institutional theory [6,7,8], but the lack of reference to organizational design seems more challenging when discussing green innovation in a digital context [5,9]. Hence, our study incorporates organizational changes (i.e., firms’ digital transformation) and external contingencies (i.e., regulatory pressure and international opportunities) to bridge structural contingency theory and the digital perspective in the framework of firms’ green innovation. 

In the last decade, the burgeoning digital economy and its related digital tools have phenomenally spurred organizations into an accelerated digital transformation, which has triggered frequent organizational change and, hence, innovation [10,11]. Therefore, digital transformation is viewed as organizational change in strategy, structure, supply chains, and marketing enabled by firms’ widespread use of digital technologies, aiming at constructing a new digital business model that contributes to the creation and appropriation of greater value [12,13]. It is naturally connected to the topic of innovation. As a type of innovation that concerns environmental issues, green innovation refers to the incorporation of novel green technologies into production to mitigate negative impacts on the environment and improve the productive use of energy [3,14]. Most extant studies have focused on the direct effects of firm capabilities and knowledge on green innovation [15,16,17], whereas little research has covered how organizational transformation shapes corporate green innovation. There is a consensus that digital transformation is a vital conduit for organizational decentralization and management efficiency enhancement [12,18,19,20], and that it fundamentally promotes diversified innovation, as postulated in structural contingency theory [21,22]. Therefore, our study tracks the logic and proposes that a firm’s digital transformation will significantly facilitate its green innovation.

Recognizing the classic contingency theory, which holds that organizations have a proper “fit” with environmental challenges and opportunities [23], we suggest that some external contingency factors should be investigated. Our purpose is to figure out the market conditions under which corporate green innovation is more or less sound as affected by digital transformation. As previous researchers and practitioners reveal, environmental regulations can be more challenging in some developing and emerging economies [4,24]. In China, administrative policies to achieve greening goals vary by province, rendering firms less or more engaged in organizational changes that impact green innovation. This condition provides us with a chance to observe sub-national differences in environmental regulations and innovation feedback. At the supranational level, firms born in developing economies are tempted by the gains from global markets, and thus commit more to meeting environmental standards in focal countries. We expect that the effect of digital transformation is promoted when (1) firms are challenged by higher-level regulatory pressure, because organic organizational structures are increasingly required to handle the resulting task interdependence; and (2) firms perceive remunerative international opportunities, because seeing high task uncertainty evokes more efficient digital infrastructure and architecture in green innovation.

We tested our theoretical arguments on a sample of 2010 Chinese-listed firms during the 2012–2019 period and the results largely support our hypotheses. Our study makes three contributions to the literature. First, by taking a digital perspective on firms’ green innovation, our study refines the traditional non-digital setting for green innovation in the digital era. Our findings confirm that digitalization strategies accelerate firms’ adoption of greener and cleaner operations in a developing country context. Second, we creatively employ the contingency paradigm to explain how external contingencies influence organizational changes through digitalization. Exploring the task interdependence and uncertainty stemming from external challenges and opportunities, we enrich the theoretical analyses on the moderating roles of external contingencies in the discussion of corporate green innovation. Third, we elaborate on the impact of internationalization on green innovation for emerging market firms wishing to benefit from serving more foreign customers. Our study provides interesting insights by building a compound view of digital internationalization and global–local environmental responses.

## 2. Theory and Hypotheses Development

### 2.1. Literature and the Structural Contingency Perspective

With high regard for business and social sustainability, green innovation embodies firms’ response to structural, regulatory, and supranational challenges [15,25]. Numerous studies have examined firm characteristics such as knowledge-based capabilities [8,26], principal–agent arrangement [15,27], and financial interests [24,28], as well as institutional pressure imposed by stakeholders, including political pressure from governmental institutions [6,29], societal pressure from non-governmental organizations [30], and market pressure from supply chains [31], competitors [32], and customers [33]. Nevertheless, little is known about organizational infrastructure for efficient technology exploitation in green innovation, especially in the digital era.

**Figure 1:**
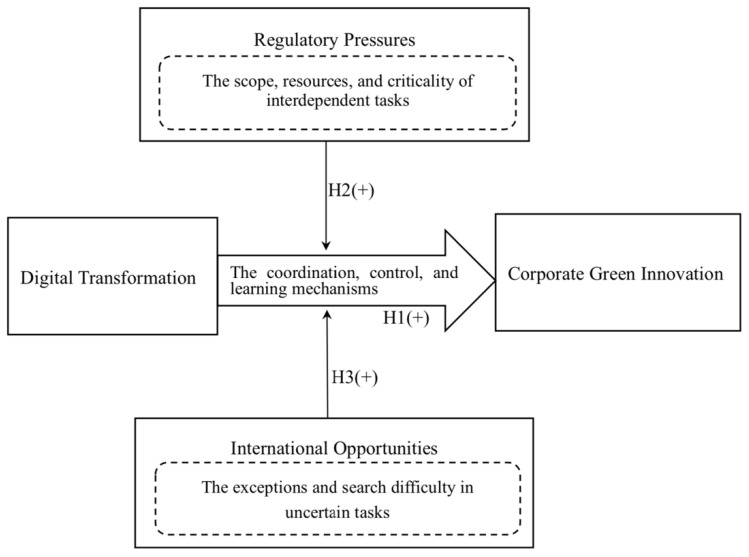
Conceptual model.

Embedded in a context of digital internationalization, more complexity may be involved in the process of new knowledge creation and diffusion to improve the efficiency and efficacy of green innovation [5,34]. What previous literature has not examined is how digital technologies reshape firms’ organizational and skill infrastructure to advance their environmental management, and what factors influence firms’ efficient adoption of digital tools for the sake of sustainable innovation. In this study, we examine the impact of digital transformation on green innovation by employing the framework of structural contingency theory. We clarify how organizations adjust to achieve greening goals through technology, in response to the moderating effects of external contingencies such as pressure and opportunities [35]. Our central argument is that digital transformation helps firms exploit green technologies more efficiently. Thus, excellence in exploiting internal organizational changes allows avoidance of environmental uncertainties [36]. This provides us with an interesting perspective to scrutinize the Chinese context, which is full of challenges and opportunities. Given that “the model of choice is never satisfied in fact and that deviations from the model accommodate the problems of introducing change” [37] (p. 72), and following the contingency paradigm of Battilana and Casciaro [38], we identify the external contingencies that influence the organizational status quo, namely, regulatory pressure from sub-national government and international opportunities from trading across foreign markets. These factors can positively moderate the relationship between internal structural contingencies (i.e., digital transformation) and corporate green innovation. We capture three general propositions considering the determinant of organizational structure: the degree of firms’ digital transformation; the task interdependence proposition (e.g., regulatory pressure); and the task uncertainty proposition (e.g., international opportunities) in our proposed conceptual model (see Figure 1).

### 2.2. Digital Transformation and Green Innovation

Driven by the assertion that “digital skills carry a general-purpose character which is important in the face of the sustainability transition that entails the creation of new activities as well as the adaptation of existing ones” [5] (p. 13), we uncover the key function of digital transformation in firms’ implementation of their environmental sustainability strategy and in defining the proper fit of organizational structure for green innovation. According to [39], digital globalization enables a growing body of modern firms to benefit from digital infrastructure and zero marginal costs. Meanwhile, digital technologies pave the way for firms to execute effective coordination and internal control and thereby embrace diversified knowledge, leading to continuous organizational changes and innovation [10,19]. Hence, we clarify two main mechanisms employed by organizations to accomplish green innovation via self-reinforcing digital transformation: the coordination and control mechanism, and the organizational learning mechanism [40].

#### 2.2.1. Coordination and Control Mechanism

Coordination and control mechanisms are well framed into corporate governance approaches, indicating that self-regulatory structures support firms’ increasing engagement in green innovation [6,27]. Structural contingency theory notes that organic structures include decentralization and extensive interactions among staff through complex intra-firm networks, whereas mechanistic structures leverage hierarchy to achieve centralization and specialization [22,41]. However, firms are more prone to innovate when organic responses arise [42]. Digital transformation involves organizational cooperation, self-discipline, and adaptability in establishing strategic goals and managing routine tasks to attain green innovation in ways that are consistent with the organic structure [43,44].

Regarding the coordination role, we find that digital transformation is adopted by firms as a key means of aligning the greening goals and behaviors of interdependent departments, and thus transcends organizational boundaries to synthesize internal and external resources [5,45]. Meanwhile, digital intelligence somehow replaces human agency and mitigates opportunism arising from the green innovation process [27], which strengthens control. Accordingly, this mechanism helps a firm to improve its energy exploitation efficiency and reduce environmental uncertainties. First, digital transformation can be set as a firm-level strategy to orchestrate different business units, and digital entrepreneurship ensures a long-term orientation of sustainable development, which drives the value creation and appropriation process [46]. The orchestration role means that digital leadership sits at the core of many task-oriented collaborations to organize value-creating interactions [47]. This enables firms to break outdated hierarchical relations and create decentralized structures to encourage value-adding activities based on extensive inter- and intra-firm information exchange behaviors related to green innovation needs [48]. Meanwhile, digital leadership continues to govern internal actors to demonstrate full compliance to achieve environmentally friendly targets. 

Second, digital capabilities (e.g., big data, artificial intelligence, the Internet of Things, and cloud computing) reshape the employee ecosystem and establish digital, energy-saving, and sustainable mindsets [49]. Responsive workers and flexible business units are automatically linked to different green tasks when embedded in the digital architecture. This helps enhance the efficacy of labor’s e-skills and human–machine interactions to foster enduring synergies between new technologies and workers. 

Furthermore, the digital infrastructure—including digitized workplaces, virtual operating systems, and decision support systems based on big data and artificial intelligence—further introduces open innovation to organizations [50]. In this way, firms adopting a digital transformation strategy are able to focus more on the interests of external stakeholders [51], with emphasis on developing modular, transparent, and adaptable structures [52]. In doing so, firms progressively achieve green innovation toward economic sustainability. Correspondingly, organizational control is implemented through these digital features, including access, behavior, output, and external relationship control, by employing district-specific procedures [53].

#### 2.2.2. Organizational Learning Mechanism

Organizational learning mechanisms enable adaptation to the technological attributes of firms’ digital transformation, where informal socialization and big data access across industry boundaries perpetually stimulate green innovation diffusion. Equipped with strong connections and data insights, organizations are more likely to provide opportunities for shared learning and high autonomy to encourage innovation [54]. Following this logic, employees with e-skills (i.e., knowledge about digital technologies) are expected to volunteer more frequently to share ideas, transfer external information into organizations, and help the organization adapt to unpredictable environments. A learning orientation in digitized organizations with flexible structures enables employees to engage more in green innovation and deal with competitive marketing forces [55].

First, digital transformation increases technological flexibility within the organization such that staff from various business units can cooperate to assimilate green technologies and actively participate in the innovation process. The phenomenon of digital transformation is regarded as a signal for businesses to integrate currently accessible knowledge into a process of thorough organizational reconstruction [12,13]. By letting “a thousand flowers bloom”, an organic structure with dispersed functions and fluid responsibilities becomes more effective under conditions of highly complicated interconnected technologies and market changes [56]. Through open innovation and task-oriented modes, digitalization can enhance organizational learning abilities and dynamic capabilities [57]. Thereby, internal knowledge through coordination and external knowledge by automatic transfer gradually accumulate and boost green innovation within organizations.

Additionally, the data-driven decision-making process shifts organizations’ learning frequency to a higher level, which, together with planned organizational changes, yields more dynamic innovation related to green technologies [3,58]. With the use of digital tools, firms can effectively recognize and capitalize on internal and external knowledge and apply what they have learned to modify product designs and daily routines to decrease environmental hazards [25]. 

In summary, firms’ digital transformation modifies workplaces and practices through coordination and control mechanisms as well as organizational learning mechanisms. Digital transformation depicts the dynamic nature of organizational structures in a digitalization context, such that green innovation levels are expected to be high. Thus, we propose:

**H1.**  *Digital transformation has a positive effect on firms’ green innovation.*

### 2.3. Moderating Effects

According to the structural contingency theory, a firm’s organic structure is enhanced when dealing with high levels of task contingency [42]. To deal with contingencies, a firm’s organic structure design is continually adjusted to decrease organizational formalities and increase structural decentralization [56], pushing a digitized organization to become more open to innovation. Hence, we categorize task contingencies into challenges and opportunities, which aptly capture the task interdependence and uncertainty in digitized workplaces [38].

#### 2.3.1. The Moderating Role of Regulatory Pressure

There are many challenges to consider; however, regulatory pressure is of primary concern [30,59,60]. In emerging economies, for example, China, regulatory authorities (e.g., sub-national governments and legislatures) may subsidize and impose taxes on firms, as well as enact laws, administrative policies, and informal norms to minimize the effects of negative impacts stemming from environmental hazards on the collaborative evolution of businesses, markets, and society [30,61]. Environmental regulations positively interact with firms’ support and operation systems that are embedded in adaptable and flexible organizational structures, and impact firms’ green technology exploitation in innovation [34,62].

Considering the underlying mechanism, we argue that when firms are challenged with high regulatory pressure, they behave more organically in terms of socialization and knowledge absorption, which facilitates the favorable effects of digital transformation on green innovation [7,38]. Higher-level socialization and knowledge absorption represent greater task interdependence, which may also signify the interconnectedness of organizational activities [63]. 

We follow Kiggundu’s [64] logic and classify task interdependence into the subdimensions of scope, resources, and criticality. From the dimension of scope, the breadth of interconnectedness of differentiated tasks significantly affects firms’ strategic choices on organizational structure [65]. In a developing country, a high regulatory pressure motivates firms to voluntarily adopt the widely recognized environmental standards in industries [66]. This requires a more decentralized coordination among various support and operation teams, including the formal and informal forms of extensive market information exchanges. In a similar vein, digitized workplaces link different subunits together more harmoniously to develop new green technologies.

Regarding the resources dimension, we contend that materials, tools, and equipment constrain organizations’ efficiency in green innovation [67]. Pursuing environmental sustainability and economic prosperity, sub-national regulatory authorities in developing economies may invest in environmental protection and treatment of environmental pollution, which arms firms with sufficient financial support, knowledgeable employees with environmental awareness and energy-saving skills, and the necessary executive power to align value chains during the greening process [68,69]. A stronger sense of reciprocity then emerges among organizations, individuals, and society, which improves the efficiency of firms’ digitalization in the pursuit of environmental sustainability.

The dimension of criticality implies that certain tasks are assigned top priority, as they affect the final outputs of an organization [64]. In a green innovation context, a high-level regulatory pressure means that more emphasis should be placed on the greening targets, which can be set as the emergent firm-level orientation. To create and apply new green technologies, all subunits are organically united to share knowledge and information and bring about positive outcomes. As Donaldson explains, “knowledge and information required for innovation are distributed among lower hierarchical levels and so decentralized decision making fosters innovation” [56] (p. 2). In other words, aggressive regulations encourage task interdependence, leading corporations to leverage digital transformation more efficiently. Thus, we propose:

**H2.**  *Regulatory pressure positively moderates the relationship between digital transformation and firms’ green innovation.*

#### 2.3.2. The Moderating Role of International Opportunities

The way firms perceive opportunities reflects their ambition to capture substantial future flows when confronting environmental uncertainty. Arising from environmental uncertainty, task uncertainty creates the need for participatory structures, forcing organizations to remain organic to cope with turbulent international situations [70]. Following Victor and Blackburn’s [71] logic, we conceive of task uncertainty as comprising two aspects: task exceptions and search difficulty. Both aspects call for a more collaborative decision-making process and, therefore, increased investment in information-processing competencies [72]. Building on the globalization context, we find that firms that rely more on international opportunities may face more task uncertainty.

Given the global–local attention situation, a lot of factors are found to be outside of emerging economy firms’ accepted wisdom when handling transnational transactions. Firms benefit from the growth in global demand, but they must simultaneously respond to varied tastes and environmental rules, including the ISO 14000 series of standards and government-initiated voluntary environmental programs across global markets [73]. Firms’ reception of diverse institutions embedded in international trade networks may differ from their long-standing understanding of home markets’ fragile institutions [74]. This, in turn, increases the task exceptions in management practices, compelling firms to focus more on precise prediction and decision systems (i.e., digital solutions) to fulfill green innovation needs in global markets. 

To seize international opportunities, firms desperately search for international buyers and benefit from the scaled economy. Consequently, they need to strengthen their formal and informal interactions with the downstream industries or consumers through global supply chains [75,76]. Moreover, local customers may think highly of the foreign sellers with “green labels” and prefer to buy goods from them [77]. Also, international firms are required to meet buyers’ (or suppliers’) expectations to reduce environmental impacts [25]. In such a situation, firms exhibit a strong commitment to the external environment (e.g., by showing great interest in ecologically sustainable strategies and practices) in focal countries, to stand out to local customers [78]. Meanwhile, existing trade partners are regarded as critical links in complicated trade networks with high potential [79]. Thus, an organization with high international opportunities is likely to exert more effort in terms of digital transformation, which strengthens cross-border business relationships, and therefore improves green innovation levels. Thus, we propose:

**H3.**  *International opportunities positively moderate the relationship between digital transformation and firms’ green innovation.*

## 3. Methods

### 3.1. Setting and Sample

We developed a panel dataset of firms listed on China’s stock exchanges (i.e., Shanghai and Shenzhen stock exchanges). As an emerging/developing economy, China provides an appropriate empirical setting to test our framework for several reasons. First, China has declared digitalization a central part of its economic growth in recent years, encouraging companies to transform their businesses digitally to remain competitive [80]. Second, the Chinese government has formulated a series of policies and programs to curb high pollution and improve environmental quality, which affects corporate green practices [81]. Third, because of the uneven regional development in institutions and markets, China has substantial sub-national disparities in the institutional environment, including environmental regulatory requirements and implementation [82]. Such variations in the intensity of environmental regulation across regions allowed us to examine the moderating roles of region-specific idiosyncrasies. Furthermore, China has been experiencing substantial growth in exports and international investments over the past two decades. Considerable variations exist in the level of internationalization among Chinese firms [83], implying that attempts to explain its moderating effect will be fruitful. Additionally, all firms listed on the stock exchanges need to disclose their annual information on strategic decision-making and organizational change. This allowed us to collect data for identifying their digital transformation status and the related impact on green innovation.

To construct our sample, we merged firm-level data on listed firms and provincial-level data from multiple sources. We began with all firms listed in China’s stock exchanges from 2012 to 2019. For green innovation, we obtained patent information from the Chinese National Intellectual Property Administration (CNIPA). For digital transformation, we relied on the annual reports of focal firms. We obtained companies’ financial and ownership information from the China Stock Market and Accounting Research (CSMAR) database. We collected information on provincial environmental regulations from the China Environmental Statistical Yearbooks. Additional regional information was obtained from the China Statistical Yearbook, China City Statistical Yearbooks, and National Economic Research Institute of China (NERI). We excluded financial companies because they are regulated entities and subject to other disclosure requirements. We also removed firms that were under special treatment by stock exchanges, given their abnormal financial status. After eliminating missing values and lagging all the independent, moderator, and control variables by one year in the regression model, the final sample used in our study comprised 8873 firm-year observations of 2010 firms.

### 3.2. Measures

#### 3.2.1. Dependent Variable

The dependent variable, green innovation (GI), is measured by the number of green patents, which is commonly used in the literature [69,84]. Patent data are collected from the CNIPA database, and patents are classified as “green” based on the World Intellectual Property Organization’s International Patent Classification (IPC) Green Inventory. The green innovations are scattered across the IPCs in numerous technical fields, including alternative energy production, transportation, energy conservation, waste management, agriculture/forestry, administrative, regulatory or design aspects, and nuclear power generation. Chinese patent law classifies patents into three types: invention, utility model, and design. Following prior studies [15,85], we focus only on invention and utility model patents because design patents are not environment-related innovations. Although patent counts have some limitations as proxies of firm innovation, since not all innovations are patented and not all patents are equally valuable, they are nevertheless directly related to organizational inventiveness. Thus, patent counts were widely adopted in previous studies to measure innovation output [6,86]. Moreover, we performed a robustness test using the number of non-self-citations per green patent as an alternative measure to assess the significance and quality of environmental innovation [6,85].

#### 3.2.2. Independent Variable

Our key independent variable was digital transformation (DT). Following previous research [87,88], we constructed an annual digital transformation index for each firm by adopting text analysis of its annual reports. First, we compiled a dictionary of keywords related to digital transformation by analyzing Chinese government documents thoroughly. Specifically, the 197 keywords used in this study could be classified into four categories: (1) application of digital technology (e.g., data mining, big data, cloud computing, block chain, machine learning, deep learning, etc.); (2) Internet-based business model (e.g., internet strategy, internet thinking, industrial Internet, electronic commerce, “Internet plus”, etc.); (3) intelligent manufacturing (e.g., artificial intelligence, industrial intelligence, intelligent logistics, cloud manufacturing, Internet of Things-enabled manufacturing, integrated manufacturing system, etc.); and (4) information system management (e.g., integrated software information system, information sharing system, information management, information network, industrial communication, etc.). Second, we used natural language processing techniques to measure the frequencies of the keywords identified in step one in the “Managerial Discussion and Analysis” (MD&A) section of corporate annual reports. We relied on the MD&A transcript because it was the section where the management narratively explains the company’s financial performance, business portfolio, managerial decisions, strategic change, organizational adjustment and so on. It provides qualitative information beyond the quantitative financial disclosures [89], and thus can reflect the organizational structure change process, such as digital transformation, in our study. Finally, we computed the index of digital transformation by dividing the keyword counts by the total word length of the MD&A transcript (in percentage).

#### 3.2.3. Moderating Variables

Our two moderating variables were regulatory pressure (RP) and international opportunities (IO). Following the example of previous studies on institutional pressure of environmental legitimacy [82,90], we measured region-specific regulatory pressure from two aspects. The first aspect was investment in environmental protection, which includes pollution prevention, ecological protection, environmental emergency management, environmental education, environmental supervision, and environmental governance. The second aspect was investment in the treatment of environmental pollution. We assumed that these two factors had equally important roles and calculated the value of *RP* via the Cobb–Douglas function:

(1) $$RP_{c,t}={\mathrm{environmental}\;\mathrm{protection}}_{c,t}^{1/2}\times {\mathrm{pollution}\;\mathrm{treatment}}_{c,t}^{1/2}$$

where $RP_{c,t}$ represents the level of regulatory pressure in province c in year t, ${\mathrm{environmental}\;\mathrm{protection}}_{c,t}$ is the percentage of environmental protection investment to GDP of province c in the year, and ${\mathrm{pollution}\;\mathrm{treatment}}_{c,t}$ is the ratio of investment in the treatment of environmental pollution to GDP. The environment-related expenses, to some extent, reflect the government’s wishes, efforts, and determination in reducing regional pollution, environmental risks, and other negative externalities. Therefore, the higher the investment, the stricter the regulatory pressure and scrutiny the firms face.

Regarding international opportunities, we used a popular variable, the ratio of foreign sales to total sales (FSTS), to capture the degree of firms’ international orientation. This variable indicates the extent to which a firm’s business depends on foreign markets. The use of FSTS as a measure of internationalization was consistent with the majority of the previous international business and management studies [91,92].

#### 3.2.4. Control Variables

We included firm- and region-level control variables used in prior studies as predictors of green innovation. At the firm level, we controlled for the effect of firm size, measured by the natural logarithm of the firm’s total assets. We controlled for firm age, calculated by the natural logarithm of the number of years since the firm’s establishment. As R&D investment was likely to affect a firm’s innovation and absorptive capability, we controlled for a firm’s R&D intensity, measured as the ratio of R&D expenditure to total sales revenue. Financial risks and debt burden could also influence a firm’s investment in green practices [92]; thus, we controlled for a firm’s financial leverage, which was defined as the ratio of total liabilities to total assets. In addition, we included management expenses, defined by the ratio of corporate management expenses to main business income, to control for managerial and operational efficiency, which helps transform resources into the desired innovation outputs [81]. Moreover, we controlled for firm performance by adding two controls (i.e., profitability and sales growth). Consistent with previous research [15], we measured firm profitability by the return on assets, which was defined as the ratio of net income to total assets. Sales growth was measured by the annual growth rate of a firm’s operating revenue. State ownership can affect a firm’s innovation efforts in environmental technologies, owing to governmental support and expectations [15]; thus, we included this variable, measured by the percentage of shares owned by the government. We also controlled for the effect of foreign ownership, which was measured as the ratio of shares owned by foreign investors.

At the regional level, firms operating in regions with higher levels of legal development might be more likely to seek green innovation to achieve a competitive advantage because of the favorable regulatory environment [6]; therefore, we controlled for provincial legal development, using the score of the sub-index “legal development” from the NERI index of marketization of China’s Provinces 2021 report [93]. We also included the provincial GDP growth rate because previous research had noted that local governments often placed more emphasis on economic growth than environmental protection to satisfy their superiors, thus influencing firms’ engagement in green innovation [94]. As firms undergoing digital transformation might rely on the Internet for their production, operations, and delivery processes, we controlled for municipal internet penetration, defined as the proportion of people who use the Internet among the total population of a city [33]. The data for GDP and the Internet were both collected from the China City Statistical Yearbooks. Finally, we included industry, year, and province dummies to control for the heterogeneity in green patenting incentives across industries, time-specific factors, and potential variations across regions respectively.

### 3.3. Statistical Model

We performed our estimation using panel negative binomial (NB) models because our dependent variable was a count variable with significant overdispersion [95]. The results of the likelihood-ratio test for the overdispersion suggested that an NB model was more appropriate than a Poisson regression model (*p* < 0.001) [96]. The Hausman test revealed no significant correlation between the firm-level fixed effects and other variables, indicating that the random effects model was preferable to the fixed effects model [97]. Therefore, we estimated the model using random effects according to the NB specification. All independent and control variables were lagged by one year to reduce the possible endogeneity issue derived from reverse causality and allow time for the digital transformation of firms to materialize. The model was specified as follows:

(2) $$E(GI_{it})=\exp(\beta_{0}+\beta_{1}\times DT_{i,t-1}+\sum_{J}\beta_{j}\times Control_{ij,t-1}+\nu_{i}+\varepsilon_{i,t})$$

(3) $$E(GI_{it})=\exp(\beta_{0}+\beta_{1}\times DT_{i,t-1}+\beta_{2}\times DT_{i,t-1}\times RP_{i,t-1}+\beta_{3}\times DT_{i,t-1}\times IO_{i,t-1}\;+\sum_{J}\beta_{j}\times Control_{ij,t-1}+\nu_{i}+\varepsilon_{i,t})$$

where $E\left(GI_{it}\right)$ is the predicted number of green patents of firm i in year t; $DT_{i,t-1}$ represents firm i’s digital transformation level in year t−1; $RP_{i,t-1}$ denotes the regulatory pressure, and $IO_{i,t-1}$ represents international opportunities. *Control* denotes a vector of control variables, as discussed in the Section 3.2.4. $\nu_{i}$ is the random effect for firm i, and exp($\nu_{i}$) follows a gamma distribution with a mean of 1 and a variance of $\alpha_{i}$, where $\alpha_{i}$ is the overdispersion parameter in the NB model. $\varepsilon_{i,t}$ is the random disturbance. We implemented the testing of H1 using Model 2. To test the moderating effect, we included interaction terms in Model 3. We estimated the models using the xtnbreg procedure with the random effects. The standard errors were bootstrapped to account for potential within-firm serial correlation. We also estimated conditional fixed effects NB models as a robustness check.

## 4. Results

### 4.1. Hypotheses Testing

We summarize the descriptive statistics and correlation coefficients for all variables in Table 1. Variance inflation factors (VIFs) shown in Table 1 were all well below the rule-of-thumb cutoff of 10, indicating that multicollinearity was not a concern for our regression analysis.

Table 2 reports the random effects NB regression results for testing our hypotheses. We included only the control variables used in Model 1. In Model 2, we estimated a model with independent and moderating variables. Then, we added the interaction term between digital transformation and regulatory pressure in Model 3, and the interaction term between digital transformation and internationalization in Model 4. Model 5 is the full model.

As shown in Model 2, Table 2, digital transformation relates positively to green innovation (DT: b=0.297, p=0.000). The results support H1. Hence, a higher level of digital transformation leads to more green innovation by firms. In addition, we found that firm size, firm age, R&D intensity, profitability, and legal development positively related to the dependent variable, green innovation, whereas management expenses and foreign ownership had a negative impact on green innovation.

To investigate the moderating effect of international opportunities, the interaction term of digital transformation and international opportunities was added (see Model 4 in Table 2). The coefficient for the interaction term was positive and significant (DT × IO: b=0.642, p=0.007). Hence, H3 was supported. Model 5 in Table 2, which includes both DT × RP and DT × IO, shows similar results. The coefficients for digital transformation and the two interaction terms were positively significant.

**Figure 2:**
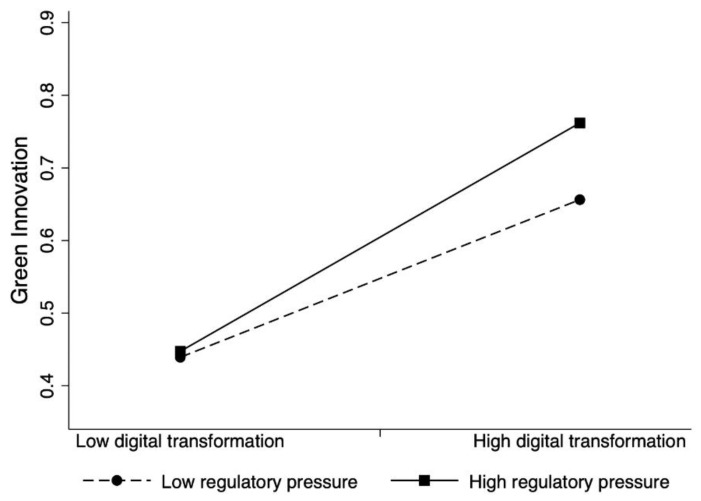
The moderation effect of regulatory pressure on the digital transformation–green innovation relationship.

**Figure 3:**
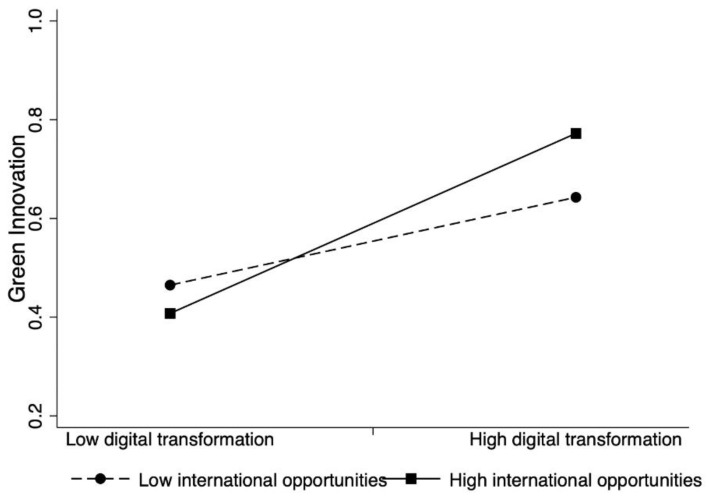
The moderation effect of international opportunities on the digital transformation–green innovation relationship.

To illustrate the results, we plotted the interactions, which are displayed in Figure 2 and Figure 3. Confirming the hypothesized moderating effects, the slope of the line for a high level of regulatory pressure is steeper than the one at a low level, and the slope of the line for a high level of international opportunities is steeper than the one at a low level. Simple slope analyses indicate that digital transformation has a strong positive association with a firm’s green innovation when the regulatory pressure is high and when the firm has a strong international orientation.

### 4.2. Robustness Tests

We conducted four sets of robustness tests to check the validity of our findings. First, although our study implements the random effects NB model, we used fixed effect estimates and re-regressed the original model to consider the fixed unobserved heterogeneity among firms [96]. Owing to the proportion of firms with few observations, the unconditional fixed effects approach was likely to produce inconsistent estimates due to the incidental parameter problem [95]. Hence, we employed an estimation with conditional fixed effects rather than an unconditional estimation. In this case, firms with green patents that are equal to zero over the whole analyzed period were eliminated because the NB fixed-effects estimator is conditional on the total number of green innovations of each firm. As Table 3 reveals, the results provided support for the robustness of our main model, as the results pertaining to our hypotheses remained qualitatively the same as those in Table 2.

Second, we tested different alternative measures for our dependent and independent variables. We used citation counts to measure green innovation instead of patent counts to capture the quality of innovation [6,98]. We also alternatively used the total number of identified keywords associated with digital transformation in the MD&A as a proxy of digital transformation. As reported in Table 4, the results were consistent with those reported in Table 2.

Third, given that the suitable lag structure of the independent variable was another estimation issue, we undertook an additional lagged specification approach. Specifically, we evaluated the model by considering two-year and three-year lags (Table 5) and the average value over three lagging years on the green innovation (unreported). The results aligned with those of the baseline specification in Table 2, suggesting that our findings remained robust in different time lags.

Finally, to address potential endogeneity in the digital transformation–green innovation relationship, following [91], we further conducted two-stage instrumental variable (IV) regressions. We employed industry-level digital transformation as the instrument, which was defined as the mean of the firm’s digital transformation level in an industry. This instrument was chosen because it was closely related to a firm’s digital transformation but had little influence on the firm’s decision on environmental innovation. The Hansen test for over-identification confirmed the IV validity and exogeneity ($\chi^{2}=1.804$, p=0.179). In the two-stage regressions, we first used the instrument to extract the exogenous component of DT. Then, in the second stage, we calculated the predicted value of DT and used it instead of DT in the NB regressions. As shown in Table 6, the second-stage results of our instrumental variable regressions remained consistent with our main results, suggesting that endogeneity did not confound the estimated effect of digital transformation and green innovation.

## 5. Discussion

Our study analyzed the role of digital transformation for corporate green innovation in a panel of Chinese firms, with a focus on the external contingencies, namely, sub-national regulatory pressure and international opportunities. Using a structural contingency approach, our findings help fill important gaps in the literature concerning the digital perspective and the drivers of firms’ green innovation, and thus offer several theoretical and practical implications, which we discuss in the following subsections.

### 5.1. Theoretical Implications

First, we investigated novel digital strategies in firms’ green innovation. Existing literature has always focused on non-digital settings, paying little attention to organizational structures when discussing the drivers of firms’ green innovation. By contrast, we argued that digital transformation can be a valid strategy to draw individuals and teams into high-quality green innovation. Since organizations adopting higher-level digital transformation are more flexible, adaptable, and decentralized, their subunits are orchestrated and controlled more organically, with enhanced learning abilities in new green technologies. Our results suggested that flexible organizational structures were effective in managing environmentally friendly business practices, which significantly encouraged the creation of new green technologies.

Second, we enhanced the conceptualization of external contingencies to apply the structural contingency approach to corporate green innovation. Digitally empowered entrepreneurial firms are exposed to massive data and are highly responsive to green tasks, nevertheless there are task contingencies that refine the efficacy of digital transformation strategies. The relevant theory categorizes task contingency as task interdependence and task uncertainty, which helped us further interpret the challenges and opportunities behind the mechanism. As task interdependence and uncertainty encourage firms to decentralize, the positive effect of firms’ digital transformation strategy on green innovation is strengthened. 

Third, we shed light on the evolutionary concept of internationalization. Although recent studies related to corporate green innovation always emphasized home-market regulatory pressure, we established a compound framework to combine regulatory pressure and opportunities for international expansion. Concerning task uncertainty, we argued that firms’ digitalization outcomes were shaped by task exceptions and search difficulties arising from the aspect of serving foreign markets. Firms’ digital strategies and business practices continuously evolved to stimulate green innovation, as more dynamism and flexibility were required to handle complexity in global marketplaces.

### 5.2. Practical Implications

Our study also has some practical implications. First, our results confirmed that a firm’s digital transformation has a positive effect on its green innovation. We provided practical insights for firms that want to improve their green innovation by implementing digital technologies for management and business purposes. Second, our results showed that the impact of digital transformation on green innovation was stronger when regulatory pressure intensified. To enhance firms’ environmental performance, governments should introduce measures to create effective institutional arrangements to encourage firms to participate in green practices. Third, we also found that the relationship between digital transformation and green innovation might be strengthened as firms have a higher level of internationalization. This finding suggests that international opportunities help firms reap more benefits from their digital transformation. Thus, firms can enjoy the unprecedented opportunities offered by digitalization and simultaneously leverage the opportunities presented by global markets, which will enable them to develop competitive capabilities and enhance their green innovation performance.

### 5.3. Limitations and Future Research Directions

Our study had some limitations that should be acknowledged. First, our study only focused on green innovation. Future research might examine the effect of digital transformation on other forms of sustainability-related innovation outcomes. Second, we only examined two types of moderators: regulatory pressure and international opportunities. Future studies could extend this research to assess additional moderators such as organizational redundancy, abilities, or market uncertainty. Third, the data are all from China and data from other countries need to be examined. We encourage future studies to duplicate and extend our findings on firms located in other emerging markets that have different institutional environments and pursue different development trajectories.

## 6. Conclusions

Over the last decade, there has been a noticeable trend toward the digitalization of business processes and practices in Chinese firms. Nonetheless, little is understood about the role of digitalization in tracing the antecedents of green innovation in corporations. Drawing on the structural contingency theory, this literature advances the research stream by developing a novel conceptual model on how organizational change (i.e., digital transformation) and external contingencies (i.e., environmental regulations and international opportunities) affect corporate green innovation in developing economies. We found that more digitized organizations were more proficient in coordination, control, and learning to accomplish green innovation. Our theoretical analyses further suggest such effects can be amplified by external contingency factors, specifically regulatory pressure and international opportunities. From the subdimensions of the scope, resources, and criticality of interdependent tasks, we found that regulatory pressure positively moderates the relationship between digital transformation and corporate green innovation. As for the aspects of exceptions and search difficulty in uncertain tasks, we propose that international opportunities also play a positive moderation role. We then tested our predictions by employing a sample of Chinese listed firms and the results supported the proposition that firms’ green innovation motivations increase with their levels of digital transformation. This positive relationship was more pronounced when firms face more environmental regulatory pressure or more international opportunities. Based on our findings in a developing country context, we find it imperative to account for digitalization strategy in driving firms to achieve green innovation in future research.

## Figures and Tables

**Table 1:** Descriptive statistics and correlations among study variables.

Variables	Mean	S.D.	VIF	1	2	3	4	5	6	7	8	9	10	11	12	13	14	15
1. GI	5.87	24.53																
2. DT	0.26	0.35	1.22	0.032														
3. RP	0.10	0.18	1.10	0.010	−0.011													
4. IO	0.13	0.20	1.04	0.008	−0.017	0.021												
5. Firm size	22.20	1.32	1.68	0.288	−0.105	0.063	−0.073											
6. Firm age	2.80	0.34	1.10	−0.044	−0.029	0.143	−0.063	0.152										
7. R&D intensity	4.58	5.22	2.24	−0.016	0.386	−0.045	−0.002	−0.255	−0.136									
8. Financial leverage	0.41	0.20	1.83	0.123	−0.159	0.025	−0.075	0.542	0.217	−0.295								
9. Management expenses	0.11	0.09	2.18	−0.025	0.126	−0.008	−0.008	−0.211	−0.010	0.703	−0.145							
10. Profitability	0.04	0.07	1.24	0.000	0.023	−0.018	−0.018	−0.036	−0.079	0.004	−0.268	0.161						
11. Sales growth	0.46	9.79	1.01	−0.002	−0.006	−0.003	−0.005	−0.006	0.006	−0.011	0.007	0.002	0.000					
12. State ownership	0.03	0.11	1.06	0.015	−0.072	−0.025	−0.069	0.168	−0.018	−0.082	0.097	−0.046	0.004	0.029				
13. Foreign ownership	0.01	0.06	1.03	−0.023	−0.012	−0.006	0.106	−0.066	−0.075	−0.003	−0.095	−0.006	0.068	−0.001	−0.022			
14. Legal development	9.47	2.81	1.28	0.058	0.200	0.022	0.102	0.017	0.067	0.095	−0.095	0.027	0.026	−0.009	−0.105	0.030		
15. GDP growth rate	7.75	1.46	1.22	−0.061	−0.184	−0.262	−0.059	−0.135	−0.254	−0.016	0.031	−0.038	0.054	0.015	0.122	0.016	−0.411	
16. Internet penetration	0.39	0.19	1.27	0.022	0.064	0.140	0.029	0.009	0.040	0.059	−0.032	0.044	0.019	−0.004	−0.031	0.027	0.282	−0.167

Note: All correlations with absolute value greater than 0.016 are significant at the α = 0.05 level.

**Table 2:** Regression results of random effects negative binomial models.

	Model 1	Model 2	Model 3	Model 4	Model 5
Main variables					
DT		0.297 ***	0.175 **	0.220 ***	0.146 *
		(0.054)	(0.062)	(0.062)	(0.067)
Interactions					
DT× RP			0.503 **		0.387 *
			(0.192)		(0.196)
DT× IO				0.642 **	0.537 *
				(0.239)	(0.243)
Moderator variables					
RP		0.143 *	0.033	0.136 *	0.057
		(0.066)	(0.080)	(0.066)	(0.08)
IO		−0.044	0.013	−0.214 ^+^	−0.148
		(0.099)	(0.098)	(0.119)	(0.118)
Control variables					
Firm size	0.403 ***	0.393 ***	0.393 ***	0.390 ***	0.394 ***
	(0.021)	(0.021)	(0.021)	(0.021)	(0.021)
Firm age	0.482 ***	0.435 ***	0.430 ***	0.440 ***	0.433 ***
	(0.065)	(0.066)	(0.067)	(0.066)	(0.067)
R&D intensity	0.031 ***	0.028 ***	0.030 ***	0.028 ***	0.031 ***
	(0.005)	(0.005)	(0.005)	(0.005)	(0.005)
Financial leverage	0.188	0.234 ^+^	0.128	0.230 ^+^	0.141
	(0.129)	(0.129)	(0.130)	(0.129)	(0.131)
Management expenses	−0.698 ***	−0.703 *	−0.720 *	−0.709 *	−0.704 *
	(0.282)	(0.292)	(0.297)	(0.291)	(0.292)
Profitability	0.556 *	0.547 *	0.638 *	0.528 *	0.629 *
	(0.266)	(0.265)	(0.267)	(0.264)	(0.267)
Sales growth	0.000	0.000	0.000	0.000	0.000
	(0.001)	(0.001)	(0.001)	(0.001)	(0.001)
State ownership	−0.177	−0.145	−0.181 ^+^	−0.139	−0.156
	(0.108)	(0.109)	(0.109)	(0.109)	(0.109)
Foreign ownership	−1.189 ***	−1.156 ***	−1.131 ***	−1.120 ***	−1.104 ***
	(0.337)	(0.339)	(0.334)	(0.339)	(0.335)
Legal development	0.034 ***	0.030 ***	0.028 **	0.030 ***	0.027 **
	(0.009)	(0.009)	(0.009)	(0.009)	(0.009)
GDP growth rate	−0.044 ***	−0.036 **	−0.048 ***	−0.037 **	−0.047 ***
	(0.012)	(0.012)	(0.012)	(0.012)	(0.012)
Internet penetration	0.341 ***	0.289 **	0.261 **	0.292 **	0.266 **
	(0.094)	(0.095)	(0.094)	(0.095)	(0.094)
Constant	−9.955 ***	−9.708 **	−9.556 ***	−9.632 ***	−9.606 ***
	(0.497)	(0.498)	(0.499)	(0.497)	(0.499)
Year, industry, and region dummies	Included	Included	Included	Included	Included
Log likelihood	−17,279.68	−16,903.27	−16,902.69	−16,900.45	−16,900.28
$\mathrm{Wald}\;\chi^{2}$	2211.70 ***	2105.14 ***	2108.64 ***	2119.65 ***	2120.95 ***
N	8873	8873	8873	8873	8873

Note: Bootstrapped standard errors in parentheses, ^+^ *p* < 0.10, * *p* < 0.05, ** *p* < 0.01, *** *p* < 0.001 (two-tailed tests).

**Table 3:** Robustness test—conditional fixed effects negative binomial models.

	Model 1	Model 2	Model 3	Model 4	Model 5
Main variables					
DT		0.282 ***	0.138 *	0.171 *	0.132 ^+^
		(0.061)	(0.069)	(0.072)	(0.076)
Interactions					
DT× RP			0.404 *		0.330 ^+^
			(0.192)		(0.194)
DT× IO				0.510 *	0.422 ^+^
				(0.256)	(0.255)
Moderator variables					
RP		0.099	0.040	0.096	0.066
		(0.064)	(0.079)	(0.065)	(0.079)
IO		−0.178	−0.224 ^+^	−0.324 *	−0.323 *
		(0.114)	(0.116)	(0.135)	(0.134)
Control variables					
Firm size	0.319 ***	0.305 ***	0.305 ***	0.302 ***	0.302 ***
	(0.025)	(0.025)	(0.025)	(0.025)	(0.025)
Firm age	0.749 ***	0.691 ***	0.611 ***	0.742 ***	0.770 ***
	(0.079)	(0.080)	(0.086)	(0.084)	(0.084)
R&D intensity	0.024 ***	0.021 ***	0.019 ***	0.020 ***	0.021 ***
	(0.005)	(0.005)	(0.005)	(0.005)	(0.005)
Financial leverage	−0.144	−0.098	−0.130	−0.116	−0.106
	(0.144)	(0.144)	(0.146)	(0.145)	(0.145)
Management expenses	−0.529 ^+^	−0.517 ^+^	−0.575 ^+^	−0.595 *	−0.658 *
	(0.282)	(0.286)	(0.300)	(0.286)	(0.3)
Profitability	0.455	0.449	0.496 ^+^	0.496^+^	0.497 ^+^
	(0.280)	(0.279)	(0.281)	(0.279)	(0.281)
Sales growth	−0.001	−0.001	−0.001	−0.001	−0.001
	(0.001)	(0.001)	(0.001)	(0.001)	(0.001)
State ownership	−0.104	−0.075	−0.014	−0.024	−0.032
	(0.118)	(0.119)	(0.122)	(0.119)	(0.119)
Foreign ownership	−0.991 **	−0.950 *	−0.910 *	−0.886 *	−0.904 *
	(0.371)	(0.374)	(0.378)	(0.377)	(0.377)
Legal development	0.038 ***	0.035 ***	0.017^+^	0.020 *	0.012
	(0.009)	(0.009)	(0.009)	(0.010)	(0.009)
GDP growth rate	−0.046 ***	−0.041 **	−0.014	−0.024^+^	−0.019
	(0.012)	(0.013)	(0.014)	(0.013)	(0.013)
Internet penetration	0.284 ***	0.246 *	0.180 ^+^	0.182^+^	0.237 *
	(0.100)	(0.101)	(0.100)	(0.104)	(0.103)
Constant	−8.644	−8.257	−7.811 *	−8.252 *	−8.286
	(0.573)	(0.576)	(0.593)	(0.582)	(0.582)
Year dummies	Included	Included	Included	Included	Included
Log likelihood	−10,417.81	−10,411.75	−10,411.15	−10,411.06	−10,410.61
$\mathrm{Wald}\;\chi^{2}$	976.90 ***	988.14 ***	990.07 ***	990.20 ***	991.78 ***
N	7203	7203	7203	7203	7203

Note: Bootstrapped standard errors in parentheses, ^+^ *p* < 0.10, * *p* < 0.05, ** *p* < 0.01, *** *p* < 0.001 (two-tailed tests).

**Table 4:** Robustness test—alternative measurements.

	Alternative Measurement of Green Innovation				Alternative Measurement of Digital Transformation			
	Model 1	Model 2	Model 3	Model 4	Model 5	Model 6	Model 7	Model 8
Main variables								
DT	0.271 ***	0.205 ***	0.288 ***	0.219 ***	0.101 ***	0.074 ***	0.074 ***	0.053 ***
	(0.030)	(0.032)	(0.034)	(0.035)	(0.014)	(0.015)	(0.016)	(0.017)
Interactions								
DT× RP		0.233 **		0.169 *		0.105 *		0.102 *
		(0.082)		(0.082)		(0.045)		(0.046)
DT× IO			0.302 *	0.263 *			0.129 *	0.132 *
			(0.131)	(0.132)			(0.065)	(0.064)
Moderator variables								
RP	0.117 ***	0.044	0.068 *	0.078 *	0.143 *	−0.178	0.116	−0.207
	(0.027)	(0.032)	(0.027)	(0.033)	(0.066)	(0.158)	(0.066)	(0.16)
IO	−0.196 ***	−0.172 **	−0.274 ***	−0.274 ***	−0.062	0.005	−0.497 *	−0.464 *
	(0.059)	(0.057)	(0.071)	(0.071)	(0.098)	(0.098)	(0.232)	(0.231)
Control variables	Included	Included	Included	Included	Included	Included	Included	Included
Year, industry, and region dummies	Included	Included	Included	Included	Included	Included	Included	Included
Log likelihood	−23,180.37	−23,179.27	−23,180.07	−23,178.85	−16,902.10	−16,901.99	−16,900.86	−16,900.78
$\mathrm{Wald}\;\chi^{2}$	18,550.88 ***	18,547.39 ***	18,543.65 ***	18,539.09 ***	2109.10 ***	2109.65 ***	2113.90 ***	2114.38 ***
N	8873	8873	8873	8873	8873	8873	8873	8873

Note: Bootstrapped standard errors in parentheses, * *p* < 0.05, ** *p* < 0.01, *** *p* < 0.001 (two-tailed tests).

**Table 5:** Robustness test—lagged variables test.

	Tow-Year Lagged Variables				Three-Year Lagged Variables			
	Model 1	Model 2	Model 3	Model 4	Model 5	Model 6	Model 7	Model 8
Main variables								
DT	0.314 ***	0.190 **	0.221 **	0.139 ^+^	0.467 ***	0.265 **	0.347 ***	0.158 ^+^
	(0.063)	(0.070)	(0.071)	(0.076)	(0.076)	(0.082)	(0.085)	(0.091)
Interactions								
DT× RP		0.535 *	0.935 **	0.442 *		0.866 ^+^		0.832 ^+^
		(0.220)	(0.317)	(0.221)		(0.502)		(0.502)
DT× IO				0.772 *			1.404 ***	1.237 **
				(0.320)			(0.422)	(0.427)
Moderator variables								
RP	0.146 ^+^	−0.032	0.132 ^+^	−0.022	0.073	0.126	0.075	0.130
	(0.078)	(0.096)	(0.078)	(0.096)	(0.125)	(0.143)	(0.125)	(0.143)
IO	0.107	0.019	−0.114	−0.106	−0.012	0.026	−0.285 ^+^	−0.215
	(0.112)	(0.115)	(0.136)	(0.138)	(0.134)	(0.138)	(0.159)	(0.163)
Control variables	Included	Included	Included	Included	Included	Included	Included	Included
Year, industry, and region dummies	Included	Included	Included	Included	Included	Included	Included	Included
Log likelihood	−23,180.37	−23,179.27	−23,180.07	−23,178.85	−16,902.10	−16,901.99	−16,900.86	−16,900.78
$\mathrm{Wald}\;\chi^{2}$	18,550.88 ***	18,547.39 ***	18,543.65 ***	18,539.09 ***	2109.10 ***	2109.65 ***	2113.90 ***	2114.38 ***
N	8873	8873	8873	8873	8873	8873	8873	8873

Note: Bootstrapped standard errors in parentheses, ^+^ *p* < 0.10, * *p* < 0.05, ** *p* < 0.01, *** *p* < 0.001 (two-tailed tests).

**Table 6:** Robustness test—second-stage instrumental variable (IV) regressions.

	Model 1	Model 2	Model 3	Model 4
Main variables				
DT_hat	0.253 ***	0.127 *	0.190 **	0.119 ^+^
	(0.055)	(0.064)	(0.064)	(0.070)
Interactions				
(DT× RP)_hat		0.576 **		0.442 *
		(0.211)		(0.213)
(DT× IO)_hat			0.498 *	0.458 ^+^
			(0.242)	(0.242)
Moderator variables				
RP	0.143 *	0.016	0.140 *	0.009
	(0.066)	(0.082)	(0.066)	(0.083)
IO	−0.055	0.006	−0.192	−0.158
	(0.099)	(0.098)	(0.120)	(0.120)
Control variables	Included	Included	Included	Included
Year, industry, and region dummies	Included	Included	Included	Included
Log likelihood	−16,903.22	−16,902.80	−16,901.16	−16,900.96
$\mathrm{Wald}\;\chi^{2}$	2105.69 ***	2107.96 ***	2117.20 ***	2118.42 ***
N	8873	8873	8873	8873

Note: Bootstrapped standard errors in parentheses, ^+^ *p* < 0.10, * *p* < 0.05, ** *p* < 0.01, *** *p* < 0.001 (two-tailed tests).

## Data Availability

Data available in the chargeable databases China Security Market and Accounting Research (CSMAR) database and Chinese National Intellectual Property Administration Database.
